# Co-administered Tag-Less Toxoid Fusion 3xSTa_N12S_-mnLT_R192G/L211A_ and CFA/I/II/IV MEFA (Multiepitope Fusion Antigen) Induce Neutralizing Antibodies to 7 Adhesins (CFA/I, CS1-CS6) and Both Enterotoxins (LT, STa) of Enterotoxigenic *Escherichia coli* (ETEC)

**DOI:** 10.3389/fmicb.2018.01198

**Published:** 2018-06-05

**Authors:** Qiangde Duan, Ti Lu, Carolina Garcia, Coraima Yañez, Rahul M. Nandre, David A. Sack, Weiping Zhang

**Affiliations:** ^1^Department of Diagnostic Medicine, Pathobiology, Kansas State University College of Veterinary Medicine, Manhattan, KS, United States; ^2^Department of International Health, Johns Hopkins University Bloomberg School of Public Health, Baltimore, MD, United States

**Keywords:** ETEC (enterotoxigenic *Escherichia coli*), toxoid fusion, MEFA (multiepitope fusion antigen), diarrhea, antibody, vaccine

## Abstract

Enterotoxigenic *Escherichia coli* (ETEC) bacteria remain a leading cause of children's diarrhea and travelers' diarrhea. Vaccines that induce antibodies to block ETEC bacterial adherence and to neutralize toxin enterotoxicity can be effective against ETEC-associated diarrhea. Recent studies showed that 6xHis-tagged CFA/I/II/IV multiepitope fusion antigen (MEFA) induced broad-spectrum antibodies to inhibit adherence of the seven most important ETEC adhesins (CFA/I, CS1 to CS6) (Ruan et al., 2014a) and 6xHis-tagged toxoid fusion antigen 3xSTa_N12S_-mnLT_R192G/L211A_ (previously named as 3xSTa_N12S_-dmLT) elicited antibodies to neutralize both heat-labile toxin (LT) and heat-stable toxin (STa) produced by ETEC strains (Ruan et al., 2014b). In this study, we constructed two new genes to express tag-less toxoid fusion 3xSTa_N12S_-mnLT_R192G/L211A_ and tag-less CFA/I/II/IV MEFA and then examined immunogenicity of each tag-less protein in mouse immunization. We further combined two tag-less proteins and investigated antigen co-administration in mice. Data showed that mice immunized with tag-less 3xSTa_N12S_-mnLT_R192G/L211A_ or tag-less CFA/I/II/IV MEFA developed antigen-specific IgG antibody responses, and mice co-administered with two tag-less proteins induced neutralizing antibodies against seven adhesins and both toxins. These results indicated tag-less toxoid fusion 3xSTa_N12S_-mnLT_R192G/L211A_ and tag-less CFA/I/II/IV MEFA administered individually or combined induced neutralizing antitoxin and/or anti-adhesin antibodies, and suggested the potential application of two tag-less proteins for ETEC vaccine development.

## Introduction

Enterotoxigenic *Escherichia coli* (ETEC) strains continue to be a leading cause of diarrhea in young children in developing countries (WHO, 2006; Black et al., 2010; Kotloff et al., 2012). ETEC was estimated to cause 280 to 400 million diarrheal cases in children under 5 years of age and 100 million cases in children above 5 years annually (WHO, 2006). That results in an annual death rate of over 150,000 children and long-term consequences of stunting among children with repeated diarrhea (Mata, 1992; Guerrant et al., 2002; Niehaus et al., 2002; Lorntz et al., 2006; WHO, 2006; Black et al., 2010). ETEC bacteria are also the most common cause of diarrhea in children and adults traveling from developed countries to developing countries as well as civil and military personnel deployed in ETEC endemic regions (Sack et al., 1975, 2007; Sack, 1978; Black, 1990; Sanders et al., 2005).

Different ETEC strains produce immunologically heterogeneous colonization factor antigen (CFA) or coli surface antigen (CS) adhesins and one or two distinct enterotoxins. CFA adhesins initiate ETEC adherence to host epithelial cells and promote bacterial colonization in host small intestines. Enterotoxins including heat-labile toxin (LT), heat-stable toxin type Ib (STa, hSTa, or STh), and occasionally heat-stable toxin type Ia (pSTa or STp) are produced by ETEC strains isolated from diarrheal patients (Nataro and Kaper, 1998; Bölin et al., 2006). LT and ST (hSTa and pSTa) elevate intracellular cyclic AMP and cGMP levels respectively in host small intestinal epithelial cells. Toxin-mediated cAMP or cGMP elevation in epithelial cells disrupts fluid and electrolyte homeostasis, leading to fluid hyper-secretion and watery diarrhea (Nataro and Kaper, 1998).

Currently, there is no vaccine licensed for ETEC-associated children's diarrhea or travelers' diarrhea (Svennerholm, 2011; Zhang and Sack, 2015 Walker, 2015). To effectively protect against ETEC diarrhea, an ETEC vaccine needs to induce broad-spectrum antibodies to inhibit adherence of ETEC strains that produce heterogeneous adhesins and to neutralize enterotoxicity of both LT and STa toxins (Zhang and Sack, 2012; Walker, 2015). Developing such an ETEC vaccine has been proven challenging in the past (Zhang and Sack, 2012); however, progress made from recent studies suggests feasibility (Walker, 2015; Zhang and Sack, 2015). Genetic fusions of an STa toxoid and a LT mutant monomer (mnLT) were demonstrated to induce neutralizing antibodies against both toxins (Zhang et al., 2010, 2013; Liu et al., 2011; Ruan et al., 2014b). Differed from the native or mutant AB_5_ holotoxin-structured LT, mnLT was created by fusing a mutated LT_A_ domain to one LT_B_ domain as a single peptide (1A-1B). Among the toxoid fusions examined, 3xSTa_N12S_-mnLT_R192G/L211A_ (previously named as 3xSTa_N12S_-dmLT) was found optimal in inducing neutralizing anti-STa antibodies (Ruan et al., 2014b; Nandre et al., 2016a,c). Toxoid fusion 3xSTa_N12S_-mnLT_R192G/L211A_ is composed of three copies of STa toxoid STa_N12S_ and one copy of a mutated LT monomer. This LT monomer is labeled mnLT_R192G/L211A_ because it carries one A subunit and one B subunit as monomeric peptide and has the LT_A_ domain mutated at the 192th and the 211th residues. It was also revealed that structure-defined CFA/I/II/IV MEFA (multiepitope fusion antigen), a chimeric peptide that uses a backbone protein to carry and to present epitopes of the seven most important ETEC adhesins [CFA/I, CFA/II (CS1, CS2, CS3), CFA/IV (CS4, CS5, CS6)], elicited antibodies that broadly inhibited *in vitro* adherence of ETEC and *E. coli* strains expressing these seven CFA adhesins (Ruan et al., 2014a; Duan et al., 2017b). However, like toxoid fusion 3xSTa_N12S_-mnLT_R192G/L211A_, CFA/I/II/IV MEFA protein carried a tag of six histidine residues (6xHis) at the N-terminus. The 6xHis-tag from protein expression vector to serve the purpose of protein affinity purification may alter biochemical and antigenic property of recombinant proteins (Wu and Filutowicz, 1999). The 6xHis-tag carried by a recombinant protein recognizes anti-histidine antibodies, often used to confirm expression and extraction of recombinant proteins. On the other hand, 6xHis-tag may also induce anti-histidine antibodies. Since histidine is an essential amino acid for human health, this brings up a regulatory concern that induced anti-histidine antibodies could cause adverse effects to vaccines. Therefore, 6xHis-tagged toxoid fusion and CFA/I/II/IV MEFA proteins are not considered optimal for ETEC vaccine development.

Toxoid fusion 3xSTa_N12S_-mnLT_R192G/L211A_ and CFA/I/II/IV MEFA become more desirable for ETEC vaccine development if they do not carry the 6xHis-tag and induce neutralizing antitoxin or anti-adhesin antibodies. In this study, we first reconstructed the toxoid fusion gene and the CFA/I/II/IV MEFA gene to express tag-less proteins, we then examined tag-less 3xSTa_N12S_-mnLT_R192G/L211A_ and tag-less CFA/I/II/IV MEFA for antitoxin and anti-adhesin immunogenicity in mouse immunization. Moreover, with individual protein immunogenicity confirmed, we combined the tag-less CFA/I/II/IV MEFA and the tag-less 3xSTa_N12S_-mnLT_R192G/L211A_ for mouse co-administration, examined induced antibodies for neutralization activities against enterotoxicity and bacteria adherence, and evaluated the potential application of two tag-less proteins for ETEC vaccine development.

## Materials and methods

### *E. coli* bacterial strains

ETEC and *E. coli* strains used for extraction of fimbrial adhesins and *in vitro* antibody adherence inhibition assays (Ruan et al., 2014a) are listed in Table 1. 6xHis-tagged CFA/I/II/IV MEFA strain 9175 (Ruan et al., 2014a) and 6xHis-tagged toxoid fusion 3xSTa_N12S_-mnLT_R192G/L211A_ strain 9331 (Ruan et al., 2014b) were used as the template to PCR amplify tag-less CFA/I/II/IV MEFA gene and tag-less toxoid fusion gene respectively. Vector pET28a (Novagen, Madison, WI) was used to clone each tag-less gene; *E. coli* strain BL21-CodonPlus (GE Healthcare, Piscataway, NJ) was used to express tag-less proteins.

**Table 1:** *Escherichia coli* strains and plasmids used in the study.

	**Relevant properties**	**Sources**
**STRAINS**		
*E. coli* BL21	*B F^−^, omp*T, *hsd*S (${\text{r}}_{\text{B}}^{-}$, ${\text{m}}_{\text{B}}^{-}$), *gal, dcm*.	GE Healthcare
H10407	O78:H11; CFA/I, LT, STa	Johns Hopkins Univ.
EL 392-75	O6:H16; CS1/CS3, LT, STa	Johns Hopkins Univ.
UM 75688	CS5/CS6, LT, STa	Johns Hopkins Univ.
E106 (E11881/9)	CS4/CS6, LT, STa	Univ. of Gothenburg
E116 (E19446)	CS3, LT, STa	Univ. of Gothenburg
2423, ETP98066	CS6, LT, STa	Washington Univ.
THK38/pEU405	CS1	Emory University
DH5α/pEU588	CS2	Emory University
9175	“6xHis-tagged CFA/I/II/IV MEFA + pET28a” in BL21	Ruan et al., 2014a
9331	“6xHis-tagged 3xSTa_N12S_-mnLT_R192G/L211A_ + pET28a” in BL21	Ruan et al., 2014b
9471	“tag-less 3xSTa_N12S_-mnLT_R192G/L211A_ + pET28a” in BL21	This study
9472	“tag-less CFA/I/II/IV MEFA + pET28a” in BL21	This study
**PLASMIDS**		
pCFA/I/II/IV	6xHis-tagged CFA/I/II/IV MEFA in pET28a (NheI/EagI)	Ruan et al., 2014a
p3xSTa_N12S_-mnLT_R192G/L211A_	6xHis-tagged 3xSTa_N12S_-mnLT_R192G/L211A_ in pET28a (NheI/EagI)	Ruan et al., 2014b
p9463	tag-less 3xSTa_N12S_-mnLT_R192G/L211A_ in pET28a (NcoI/EagI)	This study
p9464	tag-less CFA/I/II/IV MEFA in pET28a (NcoI/EagI)	This study

### Tag-less CFA/I/II/IV MEFA and tag-less 3xSTa_N12S_-mnLT_R192G/L211A_ toxoid fusion gene cloning

PCR using primes CFANcoI-F (5′-catgccatggaaatggctagcgcagtagaggat-′3; NcoI site underlined) and T7-R (5′-tgctagttattggtcaggggt-′3) with the DNA template of CFA/I/II/IV strain 9175 (Ruan et al., 2014a) amplified tag-less CFA/I/II/IV MEFA gene. PCR with DNA of 6xHis-tagged 3xSTa_N12S_-mnLT_R192G/L211A_ recombinant strain 9331 (Ruan et al., 2014b) and primers STaNcoI-F (5′-catgccatggaaatggctagcatgaatagtagc-′3) and T7-R generated tag-less 3xSTa_N12S_-mnLT_R192G/L211A_ toxoid fusion gene. PCR products were digested with restriction enzymes NcoI and EagI (New England BioLabs, Ipswich, MA) and ligated into vector pET28a (Novagen).

### Tag-less CFA/I/II/IV MEFA and tag-less 3xSTa_N12S_-mnLT_R192G/L211A_ toxoid fusion protein expression and extraction

Recombinant tag-less CFA/I/II/IV MEFA and toxoid fusion 3xSTa_N12S_-mnLT_R192G/L211A_ proteins were expressed, extracted, and refolded as described previously (Nandre et al., 2016b). Briefly, a single colony from each recombinant bacteria strain was grown in 5 ml Lysogeny Broth (LB) overnight at 37°C. Overnight-grown bacteria were transferred to 200 ml 2x YT (2x Yeast Extract Tryptone) broth and cultured till OD reached 0.6. Bacteria were then induced with isopropyl-1-thio-β-D-galactoside (IPTG; 1 mM) for 4 h, collected by centrifugation, and lysed in B-PER bacterial protein extraction reagent (in phosphate buffer; Pierce, Rockford, IL). Bacterial lysates were collected to purify inclusion body proteins using lysozyme (200 μg/ml) and 1:10 diluted B-PER by following the manufacturer's protocol (B-PER; Pierce). Extracted inclusion body proteins suspended in denaturing buffer were further solubilized with protein solubilization buffer (Novagen), refolded and dialyzed using Protein Refolding Kit by following the manufacturer's protocol (Novagen). Proteins wree stored at −80°C until use.

Refolded tag-less CFA/I/II/IV MEFA proteins and tag-less 3xSTa_N12S_-mnLT_R192G/L211A_ toxoid fusion proteins (20 μg; measured by Lowry method) were assessed for purity and integrity in 12% sodium dodecyl sulfate-polyacrylamide gel electrophoresis (SDS-PAGE) with Coomassie blue staining. Proteins were characterized in Western blot using mouse anti-CFA/I, rabbit anti-CT (1:3,300; Sigma, St. Louis, MO), and rabbit anti-STa (1:5,000; a gift from Dr. Donald C Robertson at Kansas State University) antisera accordingly. IRDye-labeled goat-anti-mouse or goat-anti-rabbit IgG secondary antibodies (1:5,000; LI-COR, Lincoln, NE) and LI-COR Odyssey gel image system (LI-COR) were used to detect each protein.

### Tag-less protein thermal stability assessment

Refolded tag-less toxoid fusion and CFA/I/II/IV MEFA proteins were evaluated for thermal stability at −80°, −20°, and 0–4°C, room temperature (20° –22°C), 37°C, and 50°C with a time course of 1, 2, 5, 7, 10, 14, 18, 21, 28, 35, and 42 days respectively. Protein samples exposed to different temperature settings and at different exposure lengths were examined in SDS-PAGE with Coomassie blue staining and Western blot using anti-CT or anti-CFA/I antiserum.

### Mouse intraperitoneal (IP) immunization with tag-less CFA/I/II/IV MEFA or tag-less 3xSTa_N12S_-mnLT_R192G/L211A_ toxoid fusion protein

Four groups (six mice per group) of 8-week-old female BALB/c mice (Charles River Laboratories International, Inc., Wilmington, MA) were used in immunization study. Mice in the first group were each IP injected with 200 μg tag-less CFA/I/II/IV MEFA refolded protein (200 μl). Mice in the second group were injected with 200 μg tag-less toxoid fusion 3xSTa_N12S_-mnLT_R192G/L211A_ protein (200 μl). Two microgram dmLT (double mutant holotoxin-structured AB_5_ LT, LT_R192G/L211A_; provided by WRAIR through PATH), in 100 μl, was used as the adjuvant for both immunization groups. Each immunized mouse received two booster injections with the same dose of the primary at the interval of 2 weeks. A third group IP immunized with 2 μg dmLT adjuvant alone (100 μl; referred as dmLT immunized group), and a fourth group without injection (referred as the control group to measure baseline mouse immune responses) were included. All mice were anesthetized with CO_2_ and then exsanguinated by following AVMA Guidelines for Euthanasia of Animals (2013 Edition) 2 weeks after the second booster. Blood samples were collected from each mouse before the primary and 2 weeks after the final booster; mouse serum samples were stored at −80°C until use.

### Mouse IP co-administration with tag-less toxoid fusion 3xSTa_N12S_-mnLT_R192G/L211A_ and tag-less CFA/I/II/IV MEFA proteins

Two groups of 8-week BALB/c mice (six per group; Charles River Laboratories International, Inc.) were used in mouse co-administration study. With immunogenicity of each individual protein verified, tag-less 3xSTa_N12S_-mnLT_R192G/L211A_ toxoid fusion and tag-less CFA/I/II/IV MEFA were combined for co-administration. A group of six mice was each IP immunized with 150 μg tag-less toxoid fusion protein (100 μl) pre-mixed with 80 μg tag-less CFA/I/II/IV MEFA protein (100 μl) at a ratio of 1:1 molecule, with 2 μg dmLT adjuvant (100 μl). Immunized mice received two boosters at the interval of 2 weeks. The third group of six mice without immunization was used as the control.

### Mouse serum anti-adhesin and antitoxin IgG antibody titration

Serum samples of each mouse immunized with the tag-less toxoid fusion were titrated for anti-LT and anti-STa IgG antibodies in ELISAs described previously (Zhang et al., 2010, 2013; Ruan et al., 2014b, 2015; Nandre et al., 2016c). Mouse serum samples of the group immunized with tag-less CFA/I/II/IV MEFA were titrated for IgG antibodies to CFA/I, CS1, CS2, CS3, CS4, and CS5 (Ruan et al., 2014a, 2015). The serum samples of the co-administered mice were titrated for antitoxin and anti-adhesin IgG antibodies. Anti-CS6 IgG antibodies were not examined due to a lack of CS6 coating antigens. Briefly, 100 ng cholera toxin (CT; Sigma; CT is the homolog of LT and is commonly used as the coating antigen to titrate anti-LT antibodies) or 500 ng heat-extracted CFA/I, CS1, CS2, CS3, CS4, or CS5 fimbriae were coated to each well of 2HB (Thermo Scientific, Rochester, NY) to titrate anti-LT and anti-adhesin antibodies respectively. Ten ng STa-ovalbumin conjugates (a gift from Dr. Don Robertson at Kansas State University) were coated to each well of Costar plates (Corning Inc., Corning, NY) to titrate anti-STa IgG antibodies. Serum samples from each immunized or control mouse were two-fold diluted (from 1:100 to 1:25,600) and examined in triplicates. Horseradish peroxidase (HRP)-conjugated goat anti-mouse IgG (1:3,300; Sigma) was used as the secondary antibody, and 3,3′,5,5′-tetramethylbenzidine (TMB) Microwell Peroxidase Substrate System (2-C) (KPL, Gaithersburg, MD) was used as the substrate. IgG antibody titers were calculated from multiplications of the highest serum dilution to produce OD_405_ of >0.3 above the background by the adjusted OD (row OD_405_ subtracted by background readings), and were presented in log_10_ (Liu et al., 2011; Ruan et al., 2014b, 2015). Serum or fecal IgA antibody response was not tested.

### Mouse serum antibody neutralization against CT and STa enterotoxicity

Mouse serum samples pooled from the group IP immunized with tag-less 3xSTa_N12S_-mnLT_R192G/L211A_ alone or combined with tag-less CFA/I/II/IV MEFA were examined for *in vitro* antibody neutralization against STa and CT enterotoxicity, by using T-84 cells and EIA cAMP and cGMP kits (Enzo Life Sciences, Farmingdale, NY) as previously described (Zhang et al., 2010, 2013; Liu et al., 2011; Ruan et al., 2014b, 2015). Neutralizing anti-LT antibodies prevent CT (LT homolog) from elevating intracellular cyclic AMP (cAMP), and neutralizing anti-STa antibodies prevent STa from stimulating cGMP levels of T-84 cells. Briefly, mouse serum sample (30 μl) pooled from each immunization group or the control group (without immunization) was mixed with 10 ng CT (Sigma) or 2 ng STa (a gift from Dr. DC Robertson), in duplicates. Incubated at room temperature for 30 min, each serum/toxin mixture was transferred to T-84 cells and incubated for 3 h (CT in cAMP) or 1 h (STa in cGMP) in a CO_2_ incubator. T-84 cells were lysed; lysates were collected and measured for intracellular cGMP or cAMP levels (pmole/ml) by following the manufacturer's protocols (Enzo Life Sciences).

### Mouse serum antibody adherence inhibition against CFA/I, CS1, CS2, CS3, CS4/CS6, CS5/CS6, and CS6 adhesins

Mouse serum samples pooled from the group immunized with the tag-less CFA/I/II/IV MEFA alone or co-administered with tag-less toxoid fusion 3xSTa_N12S_-mnLT_R192G/L211A_, and the group immunized with dmLT adjuvant were examined for *in vitro* antibody adherence inhibition activities (Ruan et al., 2014a, 2015; Nandre et al., 2016b). Briefly, ETEC or *E. coli* bacteria (3.5x10^6^ CFUs; at the MOI of five bacteria per cell) (Table 1) pre-treated with 4% mannose were mixed with 20 μl mouse serum pooled from each immunization group, the group immunized with dmLT adjuvant, or the control group without immunization, in triplicates. Each mixture was incubated on a shaker (50 rpm) at room temperature for 1 h, and then was added to confluent monolayer Caco-2 cells (ATCC #HTB-37^TM^, American Type Culture Collection, Manassas, VA; 7 × 10^5^) in a 24-well tissue culture plate containing Dulbecco's modified Eagle's medium (DMEM)-20% fetal bovine serum (FBS) (Fisher Thermo Scientific, Pittsburg, PA). Incubated in a CO_2_ incubator (5% CO_2_) at 37°C for 1 h, Caco-2 cells were gently rinsed with PBS to wash off non-adherent bacteria. Caco-2 cells with adherent bacteria were dislodged by incubation with 0.5% Triton X-100 (Sigma). ETEC or *E. coli* bacteria were collected by centrifugation, suspended in 1 ml PBS, and then serially diluted. Diluted bacteria suspension samples were plated on LB agar plates. Bacteria were counted (CFUs) after 37°C overnight growth.

### Ethics statement and animal care

A total of 36 mice used in two immunization experiments in the current study were taken care by the Comparative Medicine Group (CMG) staff at the Kansas State University. All mouse immunization studies complied with the 1996 National Research Council guidelines and were approved by Kansas State University Institutional Animal Care and Use Committee (IACUC). All efforts were made to minimize mouse discomfort and distress. Mice were housed in ventilated cages (four mice per cage; cage changed weekly) on corn cob bedding at Kansas State University specific-pathogen-free small animal facility with 12-h light cycle (7 a.m.−7 p.m.) and temperature set at 72°F. Mice were fed with Purina 5001 rodent diet and watered with chlorinated Hydropac pouches. Enrichment was supplied with Nestlets and Envirodry crinkle paper, plus huts or lofts. Mouse health checks (for signs of pain, distress, inflammation at injection sites, and illness by observing activity level, appetite and physical conditions including hunched posture, ruffled fur coat, and lameness) were conducted twice a day by AALAS (American Association for Laboratory Animal Science) certified animal care staff; mice showing any clinical signs were treated by the attending veterinarian.

### Statistical analysis

Mouse serum samples were examined in triplicates (for IgG antibody titration and antibody adherence inhibition assays) or in duplicates (for antibody enterotoxicity neutralization assays). Mouse antibody titration and antibody neutralization assays were repeated two times. Differences of mouse antibody titers (in log_10_) between the control group and the immunized group were analyzed using Student *t*-test, and mouse serum antibody neutralization activities between the control group and the immunized group were analyzed using Two-Way ANOVA at a CI of 95%. A *p* < 0.05 indicated a difference was significant.

## Results

### Tag-less toxoid fusion 3xSTa_N12S_-mnLT_R192G/L211A_ and tag-less CFA/I/II/IV MEFA induced antigen-specific IgG antibody responses in the IP immunized mice

**Figure 1:**
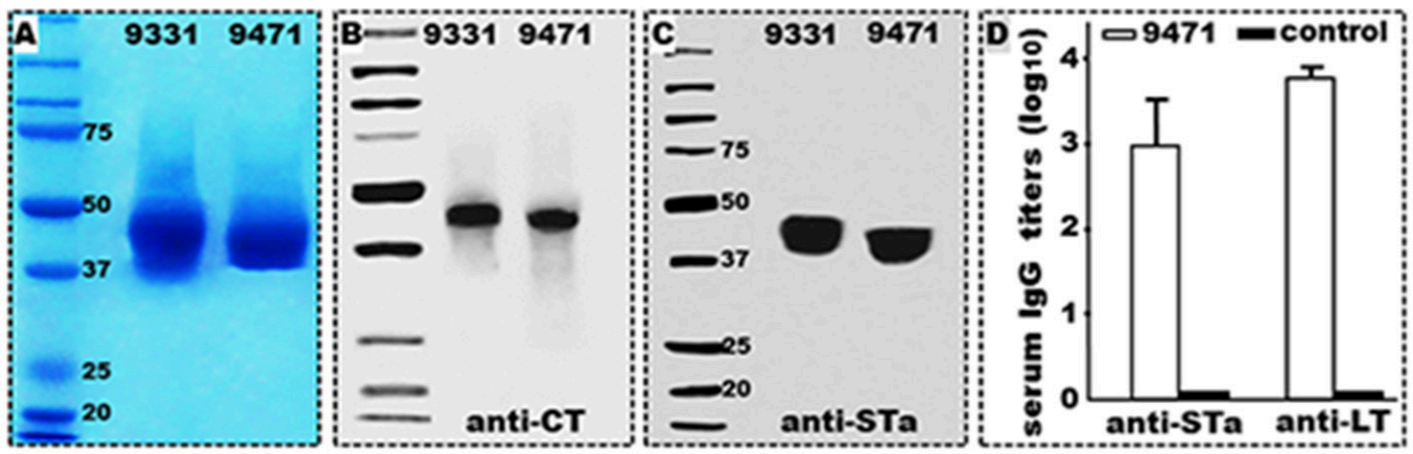
Tag-less 3xSTa_N12S_-mnLT_R192G/L211A_ (9471) recombinant protein expression, characterization and immunogenicity. **(A)** Coomassie blue staining to show refolded tag-less 3xSTa_N12S_-mnLT_R192G/L211A_ (9471) and 6xHis-tagged 3xSTa_N12S_-mnLT_R192G/L211A_ (9331) proteins electrophoresed in 12% SDS-PAGE. **(B)** Western blot detection of tag-less 3xSTa_N12S_-mnLT_R192G/L211A_ (9471) and 6xHis-tagged 3xSTa_N12S_-mnLT_R192G/L211A_ (9331) proteins with anti-CT rabbit antiserum. **(C)** Western blot detection of tag-less 3xSTa_N12S_-mnLT_R192G/L211A_ (9471) and 6xHis-tagged 3xSTa_N12S_-mnLT_R192G/L211A_ (9331) with anti-STa rabbit antiserum. **(D)** anti-STa and anti-LT IgG antibody titers (log_10_) detected from the serum samples of the mice IP immunized with tag-less 3xSTa_N12S_-mnLT_R192G/L211A_ (9471) with dmLT adjuvant or the control mice (without injection); six mice per group. Boxes and bars indicate means and standard deviations. Molecular weight marker in kDa.

DNA sequencing verified the newly cloned toxoid fusion and CFA/I/II/IV MEFA genes did not carry 6xHis-tag nucleotides. Protein extracted from tag-less 3xSTa_N12S_-mnLT_R192G/L211A_ recombinant strain 9471 was detected by rabbit anti-CT and anti-STa antibodies (Figure 1), at an estimated yield of 130–150 mg (refolded protein) per liter medium broth, a yield similar to the 6xHis-tagged 3xSTa_N12S_-mnLT_R192G/L211A_ (9331). Mice IP immunized with the refolded tag-less 3xSTa_N12S_-mnLT_R192G/L211A_ protein (with dmLT adjuvant) developed anti-STa (2.98 ± 0.5; log_10_) and anti-LT (3.77 ± 0.14) IgG antibodies (Figure 1). Mice IP immunized with 2 μg dmLT adjuvant developed anti-LT IgG antibodies (3.1 ± 0.1) which were lower than mice immunized with the toxoid fusion and dmLT adjuvant. No antitoxin IgG antibodies were detected from the serum samples of the mice without immunization.

**Figure 2:**
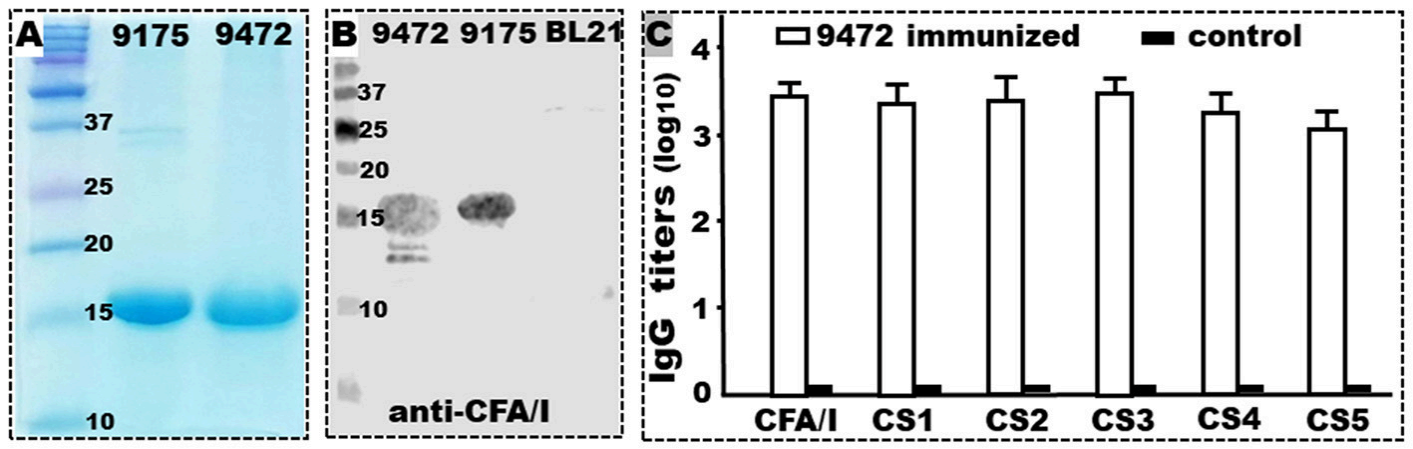
Tag-less CFA/I/II/IV MEFA (9472) protein expression, characterization, and immunogenicity. **(A)** SDS-PAGE Coomassie blue staining showed the refolded 6xHis-tagged CFA/I/II/IV MEFA (9175; ~17 kDa) and tag-less CFA/I/II/IV MEFA proteins (9472; ~15 kDa). **(B)** Western blot detection of the refolded tag-less CFA/I/II/IV MEFA (9472), 6xHis-tagged (9175) proteins, or total proteins of *E. coli* BL21 host strain, with mouse anti-CFA/I antiserum. **(C)** IgG antibody titers (in log_10_) from the serum samples of the mice IP immunized with the tag-less CFA/I/II/IV MEFA (9472; open box) or the control group (solid box; without injection); dmLT was used as the adjuvant; six mice per group. Boxes and bars indicate means and standard deviations (log_10_). Molecular weight marker in kDa.

Tag-less CFA/I/II/IV MEFA protein was expressed by recombinant strain 9472. The MEFA protein extracted from strain 9472 induced antigen-specific IgG antibody responses in the IP immunized mice (Figure 2). Tag-less CFA/I/II/IV MEFA protein was extracted at a yield of about 130–150 mg (after refolding) per liter culture broth, which was similar to the yield of 6xHis-tagged MEFA protein extracted from strain 9175. Like the 6xHis-tagged MEFA protein, the tag-less MEFA protein was recognized by mouse anti-CFA/I antiserum (Figure 2B).

The serum samples of the mice IP immunized with the refolded tag-less CFA/I/II/IV MEFA protein (with dmLT adjuvant) were detected IgG antibodies specific to CFA/I (3.5 ± 0.15), CS1 (3.4 ± 0.25), CS2 (3.4 ± 0.27), CS3 (3.5 ± 0.20), CS4 (3.3 ± 0.23), and to CS5 (3.1 ± 0.20) (Figure 2C). No IgG antibodies specific to these adhesins were detected from the serum samples of the control mice (without immunization) or the mice immunized with dmLT adjuvant.

### Tag-less toxoid fusion protein 3xSTa_N12S_-mnLT_R192G/L211A_ and tag-less CFA/I/II/IV MEFA exhibited thermal stability

**Figure 3:**
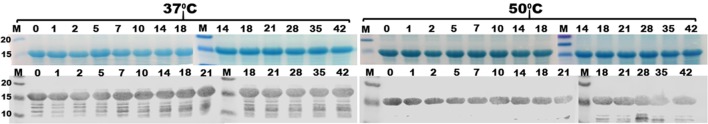
Protein thermal stability assessment of refolded tag-less CFA/I/II/IV MEFA (9472). Tag-less CFA/I/II/IV MEFA protein was characterized by SDS PAGE Coomassie blue staining **(top)** and Western blot using anti-CFA/I antiserum **(bottom)**, after exposure at 37 or 50°C for 1, 2, 5, 7, 10, 14, 18, 21, 28, 35, or 42 days. M, molecular weight marker (kDa).

No apparent protein degradation was observed from either protein exposed at the −80°, −20°, and 0–4°C, or the room temperature for at least 6 weeks, based on SDS-PAGE Coomassie blue staining and Western blot. There was no noticeable protein degradation for the tag-less CFA/I/II/IV MEFA protein stored at 37°C for 6 weeks or 50°C up to 4 weeks (Figure 3). However, noticeable degradation was observed from the tag-less toxoid fusion protein 3xSTa_N12S_-mnLT_R192G/L211A_ after 1 week at 37°C or 1–2 days at 50°C.

### The serum samples from the mice immunized with tag-less toxoid fusion 3xSTa_N12S_-mnLT_R192G/L211A_ showed neutralization activities against CT and STa enterotoxicity

**Figure 4:**
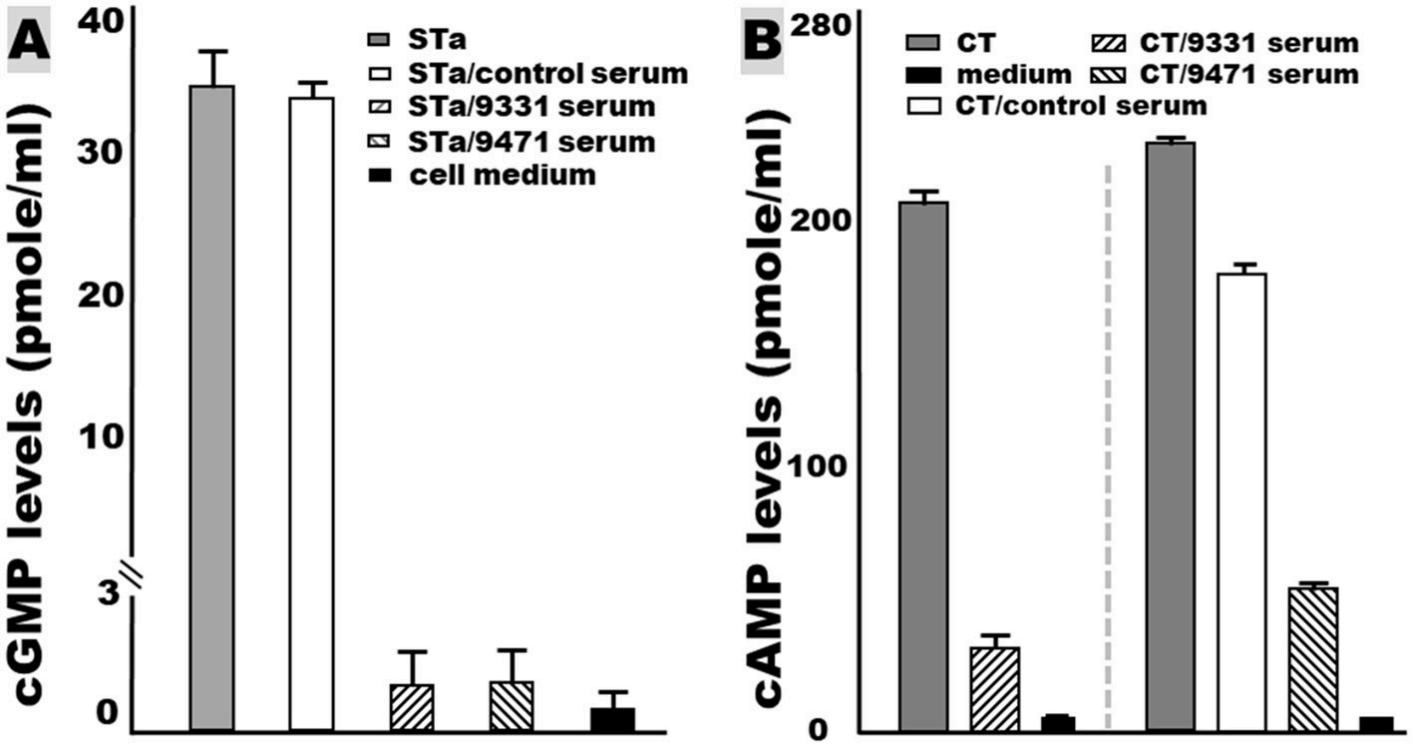
Mouse serum antibody *in vitro* neutralization activities against STa and CT enterotoxicity, measured with T-84 cells and a cGMP or a cAMP EIA kit (Enzo Life Sciences). **(A)** The serum samples of the mice IP immunized with tag-less toxoid fusion 3xSTa_N12S_-mnLT_R192G/L211A_ (9471) or 6xHis-tagged 3xSTa_N12S_-mnLT_R192G/L211A_ (9331) prevented STa toxin from elevating intracellular cyclic GMP in T-84 cells, whereas serum from the control mice (without injection) did not prevent STa from stimulating cGMP in T-84 cells. **(B)** The serum samples from the mice IP immunized with tag-less 3xSTa_N12S_-mnLT_R192G/L211A_ (9471) or 6xHis-tagged 3xSTa_N12S_-mnLT_R192G/L211A_ (9331) prevented CT toxin from elevating intracellular cyclic AMP in T-84 cells.

The serum samples of the mice IP immunized with tag-less 3xSTa_N12S_-mnLT_R192G/L211A_ showed neutralizing activities against enterotoxicity of STa and cholera toxin (CT, a homolog of LT) (Figure 4). The intracellular cGMP levels (to measure STa enterotoxicity) in the T-84 cells incubated with 2 ng STa mixed with the immunized serum samples were 1.46 ± 1.5 (pmole/ml). Those cGMP levels were significantly lower than the cGMP levels in the T-84 cells incubated with STa alone (35.3 ± 2.5; *p* < 0.01) or STa mixed with the control mouse serum samples (34.5 ± 0.9; *p* < 0.01; Figure 4A). The baseline cGMP in T-84 cells was 0.44 ± 0.02 pmole/ml.

The intracellular cAMP levels (to measure LT or CT enterotoxicity) in the T-84 cells incubated with 10 ng CT and the immunized mouse serum sample were 65.7 ± 2.2 (pmole/ml). These cAMP concentrations were significantly different from the cAMP in the T-84 cells incubated with CT alone (235.3 ± 0.98 pmole/ml; *p* < 0.01) or CT and the serum of mice without immunization (175 ± 1.4 pmole/ml; *p* < 0.01; Figure 4B).

### The serum samples of the mice immunized with tag-less CFA/I/II/IV MEFA protein inhibited adherence of ETEC or *E. coli* bacteria expressing CFA/I, CS1, CS2, CS3, CS4/CS6, CS5/CS6, or CS6 adhesin

Mouse serum samples from the group immunized with tag-less CFA/I/II/IV MEFA (9472) significantly inhibited the adherence of ETEC or *E. coli* bacteria expressing CFA/I, CS1, CS2, CS3, CS4/CS6, CS5/CS6, or CS6 adhesin to Caco-2 cells, compared to the control mouse (without injection) serum samples (Table 2).

**Table 2:** Results from *in vitro* antibody adherence inhibition assays by using the serum samples of the mice IP immunized with tag-less CFA/I/II/IV MEFA (9472) alone, co-administered with the tag-less CFA MEFA and tag-less toxoid fusion 3xSTa_N12S_-mnLT_R192G/L211A_ (9471), with 2 μg dmLT adjuvant, or the control group (without immunization).

**Mouse groups**	**Number of bacteria (%) adhered to Caco-2 cells**						
	**H10407 (CFA/I)**	**THK38/pEU405 (CS1)**	**DH5α/pEU588 (CS2)**	**E116 (CS3)**	**E106 (CS4/CS6)**	**UM75699 (CS5/CS6)**	**ETP98066 (CS6)**
Control (without immunization)	99.97 ± 7	100 ± 5	100 ± 4.6	100 ± 4.8	99.9 ± 4.5	100 ± 10.7	100 ± 2
2 μg dmLT adjuvant	86.4 ± 7.8 (*p* = 0.01)	82 ± 34.0 (*p* < 0.01)	91 ± 5.0 (*p* = 0.19)	91 ± 2.7 (*p* = 0.16)	89 ± 14.8 (*p* = 0.06)	88 ± 8.4 (*p* = 0.03)	96.6 ± 8 (*p* = 0.97)
Immunized w/ 9472 (& 2 μg dmLT)	62 ± 1.8 (*p* < 0.01)	36 ± 3.3 (*p* < 0.01)	57 ± 5.1 (*p* < 0.01)	48 ± 2.7 (*p* < 0.01)	53 ± 13.6 (*p* < 0.01)	58 ± 4.1 (*p* < 0.01)	25 ± 2.8 (*p* < 0.01)
Co-immunized w/ 9471 & 9472 (&2 μg dmLT)	52 ± 15 (*p* < 0.01)	32 ± 2.4 (*p* < 0.01)	50 ± 4.8 (*p* < 0.01)	55 ± 3.1 (*p* < 0.01)	43.3 ±1.4 (*p* < 0.01)	43 ± 3.4 (*p* < 0.01)	30 ± 1.8 (*p* < 0.01)

*ETEC or E. coli bacteria (CFU) that express CFA/I, CS1, CS2, CS3, CS4/CS6, CS5/CS6, or CS6 adhesin incubated with the serum samples of the immunized mice or the control mice (without injection) were transferred to Caco-2 cells*.

*After 1 h incubation, non-adherent bacteria were washed off, and the adherent bacteria were collected, serial-diluted, plated on LB agar plates, and counted for CFUs after overnight growth at 37°C*.

*>P-values were from the comparison between the immunized group and the control group*.

Additionally, mouse serum samples from the group immunized with 2 μg dmLT (adjuvant) alone were examined for antibody adherence inhibition activity against ETEC or *E. coli* bacteria. Compared with the serum samples of the control group (without immunization), serum of mice IP immunized with dmLT adjuvant showed a similar level of antibody adherence inhibition (in %) against CS2(DH5α/pEU588), CS3(ETEC E116), CS4/CS6(E106) and CS6(ETP98066), but greater inhibition activity against CFA/I(H10407), CS1(THK38/pEU405), and CS5/CS6(UM75699) (Table 2).

### Co-administered tag-less CFA/I/II/IV MEFA and tag-less toxoid fusion 3xSTa_N12S_-mnLT_R192G/L211A_ induced IgG antibody responses to the target adhesins and toxins

**Figure 5:**
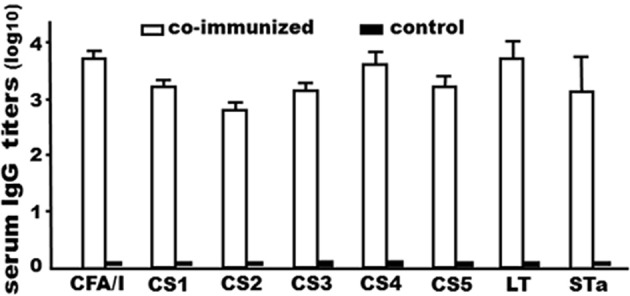
IgG antibody titers (log_10_) from the serum samples of the mice IP co-immunized with tag-less toxoid fusion 3xSTa_N12S_-mnLT_R192G/L211A_ (9471) and tag-less CFA/I/II/IV MEFA (9472), or the control mice (without injection). Six mice per group. Boxes and bars indicate means and standard deviations (log_10_).

Mice co-administered with tag-less CFA/I/II/IV MEFA (80 μg) and the tag-less toxoid fusion (150 μg) developed antibody responses to CFA/I (3.7 ± 0.18), CS1 (3.2 ± 0.13), CS2 (2.8 ± 0.11), CS3 (3.1 ± 0.16), CS4 (3.6 ± 0.24), CS5 (3.2 ± 0.18), LT (3.7 ± 0.33), and to STa (3.1 ± 0.66; in log_10_). No anti-adhesin or antitoxin IgG response was detected from the control mouse serum samples (Figure 5).

### Serum antibodies from the mice co-immunized with the tag-less toxoid fusion and tag-less CFA/I/II/IV MEFA showed neutralization activities against CT and STa enterotoxicity and adherence inhibition against CFA/I, CS1-CS6 adhesins

The serum samples of the co-immunized mice prevented STa and CT enterotoxicity from elevating cGMP and cAMP levels in T-84 cells. The cGMP levels in T-84 cells incubated with 2 ng STa exposed to the serum samples of the co-immunized group were 5.3 ± 1.1 (pmole/ml). These cGMP levels were not significantly different from the baseline cGMP levels in T-84 cells (3.7 ± 0.05 pmole/ml; *p* = 0.06), but were significantly lower than the cGMP levels in T-84 cells incubated with STa alone (69.6 ± 0.59; *p* < 0.01) or STa exposed to the control mouse serum samples (60.1 ± 3.17; *p* < 0.01).

The cAMP levels in the T-84 cells incubated with CT and the serum samples of the co-immunized mice were 68.7 ± 11.7 pmole/ml, which were significantly lower than the cAMP levels in T-84 cells incubated with CT and the control mouse serum (175 ± 1.4 pmole/ml, *p* < 0.01).

The co-immunized mouse serum samples significantly inhibited adherence of ETEC and *E. coli* bacteria expressing any of these seven adhesins to Caco-2 cells (Table 2). When Two-Way ANOVA compared with antibodies in the serum samples of the mice immunized with the CFA/I/II/IV alone, antibodies from the co-administered mice showed a similar level of adherence inhibition activities against CFA/I (*p* = 0.22), CS1 (*p* = 0.99), CS2 (*p* = 0.99), CS3 (*p* = 0.48), CS4/CS6 (*p* = 0.99), CS5/CS6 (*p* = 0.77), or CS6 (*p* = 0.21).

## Discussion

Antibodies derived from 6xHis-tagged toxoid fusion 3xSTa_N12S_-mnLT_R192G/L211A_ were demonstrated to neutralize CT and STa enterotoxicity and to protect against STa+ ETEC or LT+ ETEC diarrhea in a pig challenge model (Ruan et al., 2014b; Nandre et al., 2016a,c). Previous studies also showed that antibodies induced by 6xHis-tagged CFA/I/II/IV MEFA significantly inhibited adherence of the ETEC or *E. coli* strains expressing CFA/I, CS1 - CS6 adhesins *in vitro* (Ruan et al., 2014a, 2015). These data suggest toxoid fusion 3xSTa_N12S_-mnLT_R192G/L211A_ and CFA/I/II/IV MEFA promising antigens of ETEC vaccines. Data from the current study indicated that the newly constructed tag-less toxoid fusion 3xSTa_N12S_-mnLT_R192G/L211A_ and tag-less CFA/I/II/IV MEFA induced neutralizing antitoxin or anti-adhesin antibodies in the IP immunized mice. Moreover, two tag-less proteins can be co-administered to induce antibodies neutralizing CT and STa enterotoxicity and also inhibiting *in vitro* adherence of ETEC or *E. coli* bacteria expressing the seven most important ETEC adhesins (CFA/I, CS1-CS6). Results from this study strongly suggest the potential application of the desirable tag-less CFA/I/II/IV MEFA and 3xSTa_N12S_-mnLT_R192G/L211A_ proteins in ETEC vaccine development.

Recombinant proteins without a hydrophilic poly-his-tag are considered desirable in vaccine development (communication with Dr. Dennis Kopecko from US Food and Drug Administration). Poly-his-tag risks altering recombinant protein biochemical and antigenic properties (Wu and Filutowicz, 1999). Moreover, though one study suggested that a poly-histidine tag at the N-terminus or the C-terminus of one protein may not induce anti-histidine immunity (Sharma et al., 2009), concerns of potential adverse effects from poly-his-tag-induced antibodies to human health remain. To eliminate potential risks, we constructed new CFA/I/II/IV MEFA and 3xSTa_N12S_-mnLT_R192G/L211A_ toxoid fusion genes to express tag-less proteins. Results from this study demonstrated for the first time that tag-less CFA/I/II/IV MEFA and tag-less toxoid fusion 3xSTa_N12S_-mnLT_R192G/L211A_ recombinant proteins can be effectively expressed and extracted. Importantly, both tag-less proteins exhibited antigen-specific immunogenicity. Moreover, tag-less 3xSTa_N12S_-mnLT_R192G/L211A_ and tag-less CFA/I/II/IV MEFA were demonstrated to induce neutralizing antitoxin or anti-adhesin antibodies in immunized mice. Data from this study warrantee future downstream characterization of two tag-less proteins and may lead to the acceleration of ETEC vaccine development.

Co-administration of tag-less 3xSTa_N12S_-mnLT_R192G/L211A_ and tag-less CFA/I/II/IV MEFA induced antibodies neutralizing both toxins and inhibiting adherence of the seven most important CFA adhesins (CFA/I, CS1 to CS6), strongly indicating the potential application of two tag-less antigens in ETEC vaccine development. Because ETEC strains producing CFA/I or CS1- CS6 adhesins and LT or STa toxin cause a majority of ETEC diarrheal cases in children and travelers (Svennerholm, 2011; Zhang and Sack, 2012), an effective vaccine needs to target these seven adhesins and two toxins. A vaccine candidate carrying tag-less 3xST_N12S_-mnLT_R192G.L211A_ and tag-less CFA/I/II/IV MEFA can induce antitoxin and anti-adhesin antibodies against the seven adhesins and both toxins, thus becoming broadly protective against ETEC associated children's diarrhea and travelers' diarrhea. Additionally, a recent study demonstrated that antitoxin antibodies derived from the non-toxic tag-less 3xSTa_N12S_-mnLT_R192G/L211A_ showed little cross-reactivity with guanylin or uroguanylin (two important peptides that regulate homeostasis in host intestinal epithelial cells) (Duan et al., 2017a). That evidences this tag-less toxoid fusion is physiologically safe for human ETEC vaccine development. Moreover, co-administration with toxoid fusion did not compromise CFA/I/II/IV MEFA for inducing anti-adhesin antibodies to inhibit adherence of the seven adhesins. Antibodies derived from the CFA MEFA alone or combined with the toxoid fusion exhibited similar adherence inhibition activities. It needs to be pointed out that the current study only examined tag-less antigen immunogenicity in the IP immunized mice. Future studies using IM (intramuscular), ID (intradermal) and oral route, and perhaps with different adjuvants, can characterize better the immunogenicity of two tag-less proteins. Additionally, in this study, only mouse serum IgG antibodies were evaluated for *in vitro* neutralization activity against enterotoxicity and bacteria adherence. Future studies to examine whether antigens can induce IgA antibodies and if induced antibodies can protect against LT and STa enterotoxicity or ETEC colonization, perhaps more importantly ETEC diarrhea in animal models or a controlled human infection model (CHIM), can validate the application of these two tag-less antigens in ETEC vaccine development.

Tag-less proteins 3xSTa_N12S_-mnLT_R192G/L211A_ and CFA/I/II/IV MEFA showed thermal stability at frozen, refrigerated or room temperature for a minimum of 6 weeks. Moreover, tag-less CFA/I/II/IV protein appeared to be stable at 37°C for 6 weeks or even 50°C for up to 4 weeks. Thermal stability exhibited by these two proteins was expected since 37°C is the optimal growth temperature for *E. coli*. While tag-less CFA/I/II/IV MEFA protein remained stable for 4 weeks at 50°C, tag-less toxoid fusion 3xSTa_N12S_-mnLT_R192G/L211A_ displayed visible degradation particularly at 50°C, likely due to the heat lability of the LT_A_ domain and the LT_B_ domain. It will be much desired if an ETEC vaccine product carrying these two proteins does not need the cold chain storage or transportation. The current study pre-set a 6-week time course for protein thermal stability examination. Studies to expose proteins for a longer period and to assess protein immunogenicity can further assess protein thermal stability. Additionally, this study only used SDS-PAGE Coomassie blue staining and Western blot analyses to assess protein integrity. Future studies to evaluate protein crystallographic structure and to measure protein purity and protein refolding using methods including differential scanning calorimetry (DSC) can help to further characterize two tag-less proteins.

This study also investigated whether anti-LT antibodies derived from dmLT adjuvant assisted anti-adhesin antibodies induced by tag-less CFA/I/II/IV MEFA against ETEC or *E. coli* adherence to Caco-2 cells. In addition to adjuvanticity (dmLT) to up-immunoregulate the 6xHis-tagged toxoid fusion and to enhance antibody neutralization activity against CT (Nandre et al., 2016c), LT was shown to promote ETEC colonization in a pig model (Zhang et al., 2006). That led to a notion that anti-LT antibodies may contribute to reduce ETEC bacterial adherence or colonization (Glenn et al., 2009). The current study indicated that serum samples of the mice immunized with dmLT adjuvant exhibited no *in vitro* antibody adherence inhibition activities against CS2, CS3, CS4, and CS6 adhesins and marginally against adhesins CFA/I (100 ± 6.8 vs. 86.4 ± 7.8), CS1 (100 ± 5 vs. 82 ± 4), and CS5 (100 ± 11 vs. 87.8 ± 8.4), when compared to the control serum samples. That agrees with an early observation that serum anti-LT antibodies, if incubated with bacteria for <4 h, were unable to significantly inhibit adherence of a porcine ETEC strain to a porcine cell line (Fekete et al., 2013). Because mouse serum samples were incubated with ETEC bacteria for only 1 h in the current study, we can conclude that it was the anti-adhesin antibodies derived from the tag-less CFA/I/II/IV MEFA, not the anti-LT antibodies induced by dmLT adjuvant that inhibited bacterial *in vitro* adherence. Future *in vivo* studies especially at prolong incubation can determine any additive effect from anti-LT antibodies against ETEC colonization.

Pooled serum samples were used to examine antibody neutralization activities against LT and STa enterotoxicity and bacterial adherence from seven adhesins. Using pooled serum samples instead of individual mouse serum samples reduces the power of statistical analyses. Future studies with increased sampling sizes and using an optimized antibody neutralization assay protocol can improve assessment of neutralization activities of the antibodies derived from two tag-less proteins.

Results from this study demonstrated that tag-less CFA/I/II/IV MEFA and tag-less 3xSTa_N12S_-mnLT_R192G/L211A_ is extracted and remain immunogenic, and two tag-less proteins can be co-administered to induce neutralizing antibodies against the seven most important ETEC adhesins and LT and STa toxins. That suggests these two tag-less proteins can be potentially used to develop broadly protective vaccines and may accelerate vaccine development against ETEC associated children's diarrhea and travelers' diarrhea.

## Author contributions

WZ and DS: study design; QD, TL, CG, RN, and CY: experiment carryout; QD, TL, and WZ: data analysis; WZ: manuscript preparation.

### Conflict of interest statement

The authors declare that the research was conducted in the absence of any commercial or financial relationships that could be construed as a potential conflict of interest. The reviewer JM and handling Editor declared their shared affiliation.
